# Clinical Manifestations in 32 Cases of Mpox Infection: A Retrospective Study in a Single Dermatology Clinic in Japan

**DOI:** 10.1111/1346-8138.17789

**Published:** 2025-05-30

**Authors:** Amika Nakazawa, Tomonobu Ito, Takafumi Numata, Noriyasu Sakai, Yukari Okubo, Kazutoshi Harada

**Affiliations:** ^1^ Department of Dermatology Tokyo Medical University Tokyo Japan

**Keywords:** hepatitis B, HIV, men who have sex with men, mpox, syphilis

## Abstract

Mpox, a zoonotic orthopox virus, has re‐emerged globally since May 2022. In Tokyo, 168 cases were reported in 2023. About 20% were diagnosed in our department. In this study, we analyzed the clinical and epidemiological characteristics of 32 mpox patients diagnosed in our department in 2023. A retrospective study was conducted using medical records. All cases were confirmed for the diagnosis of mpox by PCR testing using skin samples. The six items were examined: patient background, presumed route of infection, type and site of skin eruption, clinical symptoms other than skin eruption, comorbid infectious diseases, and clinical outcome. All patients were male (median age: 39.5 years) and identified as men who have sex with men (MSM), with 30 reporting recent same‐sex sexual activity. Vesicles or small blisters and pustules were the most common eruptions, primarily on the trunk and extremities. Fever occurred in 26 cases, and penile edema was observed in four cases. The HIV‐positive rate was approximately 84%, with 53% of patients having a history of both syphilis and hepatitis B. Most cases were observed without treatment with full recovery. When seeing a patient suspected of mpox, it would be recommended to inquire about a history of sexually transmitted infections (STIs), and to test for them even if there is no prior STI history.

## 1 Introduction

Mpox virus, a zoonotic orthopox DNA virus related to smallpox, was first described in humans in 1970 in the Democratic Republic of Congo [1]. On 6th May 2022, a case of mpox was reported from the United Kingdom in a traveler who had returned from Nigeria [2]. Since then, the number of mpox cases has increased exponentially, and mpox pandemic in patients with no history of travel to endemic areas was reported [2]. There are two distinct clades of the virus: clade I and clade II. In 2022–2023, a global outbreak of mpox was caused by the clade IIb strain [3]. A total of 13 552 cases, including 76 deaths, have been reported to WHO from January 1, 2022, to November 3, 2024 [4]. In Japan, the first case of infection was reported in July 2022, and as of February 2024, 240 cases have been reported [5]. A variety of clinical symptoms such as fever and lymphadenopathy are recognized in mpox. Notably, patients suspected of mpox may visit the dermatologist, because skin lesions had occurred in 95% of patients with mpox [1]. Herein, we reported the patient backgrounds and clinical symptoms of skin lesions observed in our department.

## 2 Materials and Methods

The subjects were 32 patients diagnosed with mpox from January 1 to December 31, 2023, in our department. A retrospective study was conducted using medical record information. The study was approved by the Ethics Committee of Tokyo Medical University (approval number TS2023‐0264). The diagnosis of mpox was confirmed by PCR testing using the contents of vesicles or pustules, and vesicular lids or crusts. All PCR tests were conducted by the public health center. These tests were unable to distinguish the clades until 2024. The six items were examined: patient background, presumed route of infection, type and site of skin eruption, clinical symptoms other than skin eruption, comorbid infections, and outcome.

## 3 Results

All patients diagnosed with mpox were male. The median age was 39.5 years [range, 26–53 years], coinciding with the sexually active period. In terms of age distribution, the predominant group was individuals in their 30s and 40s. All 32 patients were men who have sex with men (MSM), and 30 of 32 patients had a history of same‐sex sexual intercourse within the past month. The remaining two patients who had no history of sexual intercourse both lived alone. One patient had episodes of frequent sauna visits, and the other attended a music event with a large crowd within a month (Table 1). A history of overseas travel within 1 month was confirmed in two cases, one of which had a history of sexual intercourse abroad.

**TABLE 1:** Demographic and Clinical Characteristics of Patients with Mpox.

Characteristic	All persons (*N* = 32)
Median age (range)	39.5 (26–53)
Ages 25–30—no. (%)	4 (13)
31–40	14 (44)
41–50	13 (41)
51–55	1 (3)
Sex or gender—no. (%)	
Male:Female	32:0 (100:0)
Men who have sex with men(MSM)—no. (%)	32 (100)
Suspected route of transmission—no. (%)	
Sexual close contact	30 (94)
Other or unknown	2 (6)
Types of skin lesion—no. (%) (including duplicates)	
Erythema	11 (34)
Erythematous papule	11 (34)
Vesicle/Pustule	30 (94)
Ulcer	7 (22)
Crust	14 (44)
Site of skin lesions—no. (%) (including duplicates)	
Only perianal area	9 (28)
Only penile	10 (31)
Both perianal area and penile	1 (3)
Trunk extremities	23 (72)
Face	10 (31)
Palms and soles	5 (16)
Reported clinical features—no. (%)	
Skin lesions	32 (100)
Fever	26 (81)
Lymphadenopathy	9 (28)
Anorectal pain	4 (13)
Penile edema	4 (13)
Pharyngitis	3 (9)
Headache	2 (6)
Fatigue	1 (3)
HIV positive—no. (%)	27 (84)
HIV negative: HIV status unknown—no. (%)	2 (6):3 (9)
Median CD4 cell count —cells/mm^3^	663.8
HIV viral load < 20 copies/mL—no./total no. with data (%)	28 (88)
Pre‐existing infections—no. (%)	
Syphilis	24 (75)
Hepatitis B	18 (56)
Medical care setting—no. (%)	
Outpatient:Inpatient	31 (97):1 (3)

**FIGURE 1:**
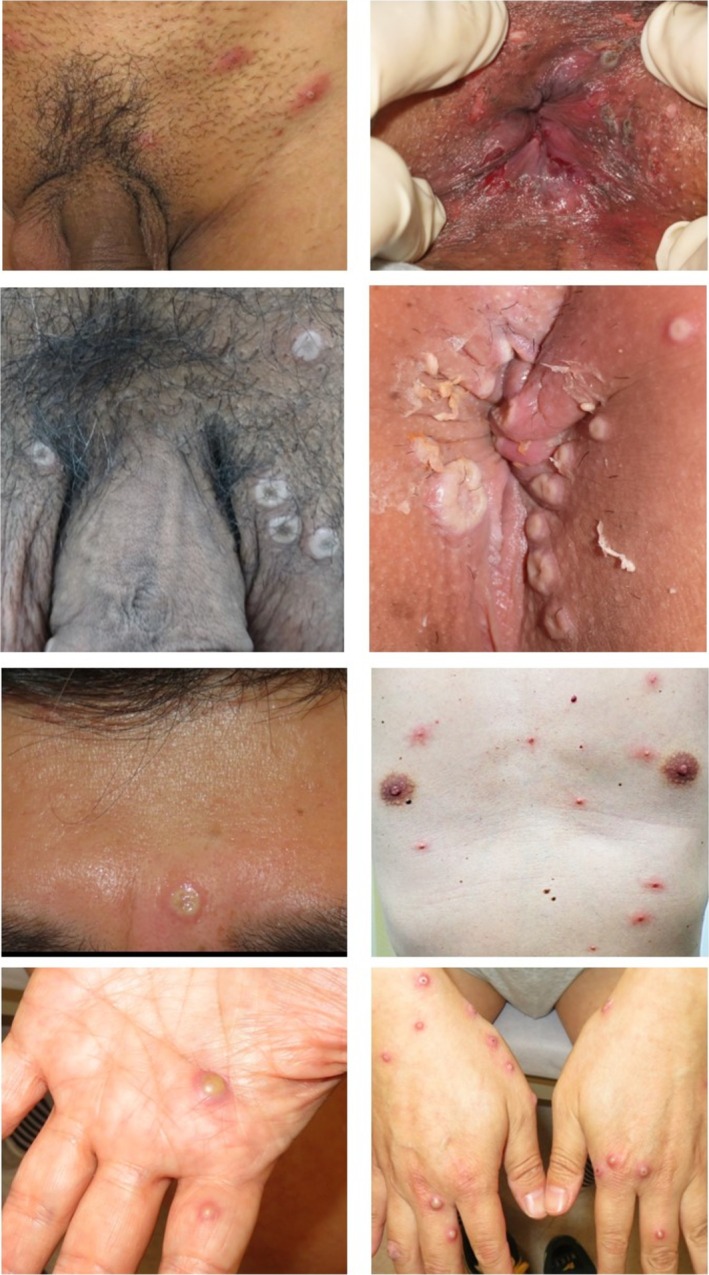
Skin lesions in patients diagnosed as mpox. Vesicles or pustules of varying sizes with depressed centers on the pubic and perianal areas. Some lesions were associated with erosions. Pustules and papules accompanied by erythema were also observed on the trunk, extremities, and face. Tense blisters were observed in the palm.

In terms of eruption type, vesicles/pustules were the most common lesions observed in 30 out of 32 cases. The size of these eruptions varied (Figure 1). In some cases, erythema or erythematous papules were associated with vesicles/pustules. A crusted vesicle/pustule or crusted ulcer was often observed. Mpox virus was detected by PCR testing in all cases of vesicles, pustules, and crusts. The most common sites of eruptions were the trunk and extremities, with 23 cases reported. The skin lesions were not always found around the genital area, even if sexual intercourse was the route of infection, suggesting that some patients had developed viremia (Figure 1). The penile edema, a rarely reported lesion in mpox infection, was observed in four cases.

**FIGURE 2:**
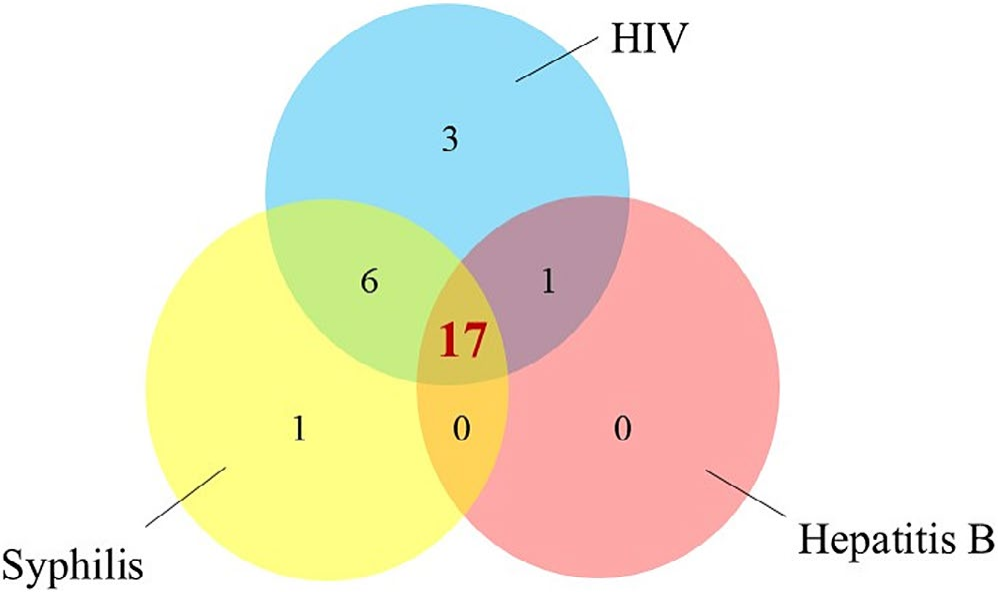
Analysis of sexually transmitted infection history in mpox patients.

The major clinical manifestations other than skin lesions included fever in 26 cases and lymphadenopathy in nine cases (Table 1). Many patients had a fever of around 38°C for 2 or 3 days before the appearance of the skin lesion. Twenty‐nine mpox patients were evaluated for HIV infection. Twenty‐seven patients were HIV‐positive; the mean value of CD4 was 663.8 cells/mm [3], and 87.5% of cases had less than 20 copies/mL of HIV viral load; most patients were well controlled for HIV. Of the 29 people tested, 24 had a history of syphilis, and of the 23 patients, serum antibody against hepatitis B was detected in 18 patients. There were 17 cases in which HIV, syphilis, and hepatitis B were all pre‐existing infections (Figure 2).

Thirty‐one patients were under observation at home and cured without treatment. Only one patient was hospitalized for a clinical trial of tecovirimat, which has already been approved in Europe for the treatment of mpox.

## 4 Discussion

In 2023, a total of 168 cases of mpox were reported in Tokyo [6]. Here, we reported 32 cases of mpox; the number of reported cases accounted for 20% of the total in Tokyo. Therefore, the characteristics of the cases presented here were thought to mimic the trends in cases across Tokyo.

Notably, all patients of mpox in this study were male and no female patients have been reported in Japan to date. As reported previously, we propose that vesicles or pustules with depressed centers on the pubic and perianal areas in MSM patients are significant findings that suggest mpox. However, there are female cases of mpox in the literature [7]; thus, mpox should be suspected in female patients with vesicles/pustules on the genital area. The reason why all mpox patients reported in Japan were male indicated that this infection spread as STI in MSM. Our hospital is the core facility for the care of HIV‐infected patients and many MSM patients visit. For this reason, a large number of mpox cases were diagnosed in our hospital.

In the present analysis, there were relatively fewer mpox patients in their 20s, the most sexually active generation, than in their 30s and 40s (Table 1), and this trend was also observed in nationwide reports in Japan [5]. The reason for this tendency remains to be elucidated. MSM may realize their own sexual preferences after they turn from their 20s to their 30s. Smallpox vaccination history was unknown in this study. The vaccine provides protection against mpox, but it was discontinued in 1977, suggesting most patients were unvaccinated.

In this study, about 94% of the Mpox cases were considered to be sexually transmitted based on medical interviews and skin lesion location. In two cases, the exact routes of infection remained to be determined. One of the two patients often used a sauna. MSM people gather at particular saunas. Floors that have not been properly cleaned or mats that have not been washed in the saunas may pose a risk of mpox infection. The other of the two patients went to a live music event before the onset of mpox. The event was held in a small space with a large audience. It has been reported that mpox is not airborne, but can be transmitted through droplets [8]. The patient might be infected with mpox through droplet infection.

In our case series, 4 cases of penile edema were included. Penile edema was considered to be due to severe local inflammation induced by mpox infection (Table 1). Since the skin eruption of mpox is usually accompanied by edema, penile edema might be the same condition. It has been reported that paraphimosis in mpox patients was a result of the exacerbation of penile edema. Urological intervention was needed in this case [9]. If dermatologists suspect circulatory failure in the glans in mpox patients, urological consultation should be considered.

In this study, the HIV‐positive rate in mpox patients was approximately 84%, with 53% of patients having a history of both syphilis and hepatitis B (Figure 2). The high prevalence of sexually transmitted infections (STIs), including HIV, in mpox patients has been reported [10]. However, few reports mentioned the co‐infection of mpox and hepatitis B. Around 10% of patients infected with HIV already have hepatitis B infection in Japan [11]. Therefore, if the patient is suspected of mpox infection, a history of STIs, including hepatitis B, should be inquired into and considered in order to perform serological tests for hepatitis B.

## Conflicts of Interest

The authors declare no conflicts of interest.
